# Stumped by a Case of Appendicitis After Appendectomy

**DOI:** 10.31486/toj.23.0098

**Published:** 2024

**Authors:** Ami Takei Yuen, Anna Suessman

**Affiliations:** ^1^ The University of Queensland Medical School, Ochsner Clinical School, New Orleans, LA; ^2^ Pediatric Emergency Department, Ochsner Clinic Foundation, New Orleans, LA

**Keywords:** *Abdominal pain*, *appendectomy*, *appendicitis*, *appendix*, *conservative treatment*, *pediatrics*

## Abstract

**Background:** Stump appendicitis—a rare, delayed complication of appendectomy—is most commonly managed with surgical exploration and stump appendectomy. Conservative management in the pediatric population is poorly characterized in the literature.

**Case Report:** We report a case of a 10-year-old male who was diagnosed with stump appendicitis and initially treated nonoperatively. He received intravenous antibiotics and supportive therapy while in the hospital, was discharged on a course of oral antibiotics, and remained asymptomatic for the following 9 weeks until he underwent an elective interval stump appendectomy. We also review the literature on this uncommon condition and treatment plan.

**Conclusion:** Considering stump appendicitis in the differential of children with history of appendectomy is imperative. Nonoperative management of stump appendicitis may be successful and beneficial in select pediatric cases compared to the standard surgical management.

## INTRODUCTION

Stump appendicitis is a rare, delayed complication of appendectomy that is characterized by inflammation of residual appendiceal tissue.^1^ Stump appendicitis is most commonly reported in adults, but several cases have been reported in children and adolescents,^2^ and surgical management remains the treatment of choice.^3^ While conservative management of stump appendicitis has been reported in adults, its role in the pediatric population is less commonly known.^4^ We present a case of stump appendicitis in a child who was managed with initial nonoperative treatment and interval appendectomy several weeks later.

## CASE REPORT

A 10-year-old male with a surgical history of laparoscopic appendectomy presented to our pediatric emergency department complaining of right-sided abdominal pain for the prior 4 days. The pain was gradual in onset and varied in severity. On the day of presentation, the pain had spread to the umbilical area. The patient had associated fever, fatigue, and nausea. His pain was relieved by lying down and aggravated by movement. He denied vomiting, abdominal distension, pain on micturition, diarrhea, or constipation. He described the current symptoms as being similar to what he experienced with an acute perforated appendicitis that was treated with an uncomplicated laparoscopic appendectomy 6 months prior. The patient had no other significant medical, family, or socioeconomic histories.

On examination, the patient was afebrile with tachycardia but otherwise nontoxic in appearance. He had significant right lower quadrant tenderness, as well as guarding with mild suprapubic and rebound tenderness. Laboratory studies showed a white blood cell count of 12.5 K/μL (reference range, 4.5-14.5 K/μL) with a left shift (absolute neutrophil count of 8.7 K/μL [reference range, 1.5-8.0 K/μL]). Abdominal x-ray demonstrated moderate stool without concerns of obstruction. Abdominal ultrasound showed a tubular structure with fecaliths and fluid collection representing a possible abscess. Abdominal and pelvic computed tomography (CT) with contrast revealed a 1.7-cm rim-enhancing mixed density mass in the region of the appendectomy stump with surrounding inflammation and potential fecalith (Figure). The patient was diagnosed with stump appendicitis based on the CT results. He was given oral acetaminophen 15 mg/kg once in the emergency department to manage his symptoms and was admitted to pediatric surgery for further evaluation and management.

**Figure.**
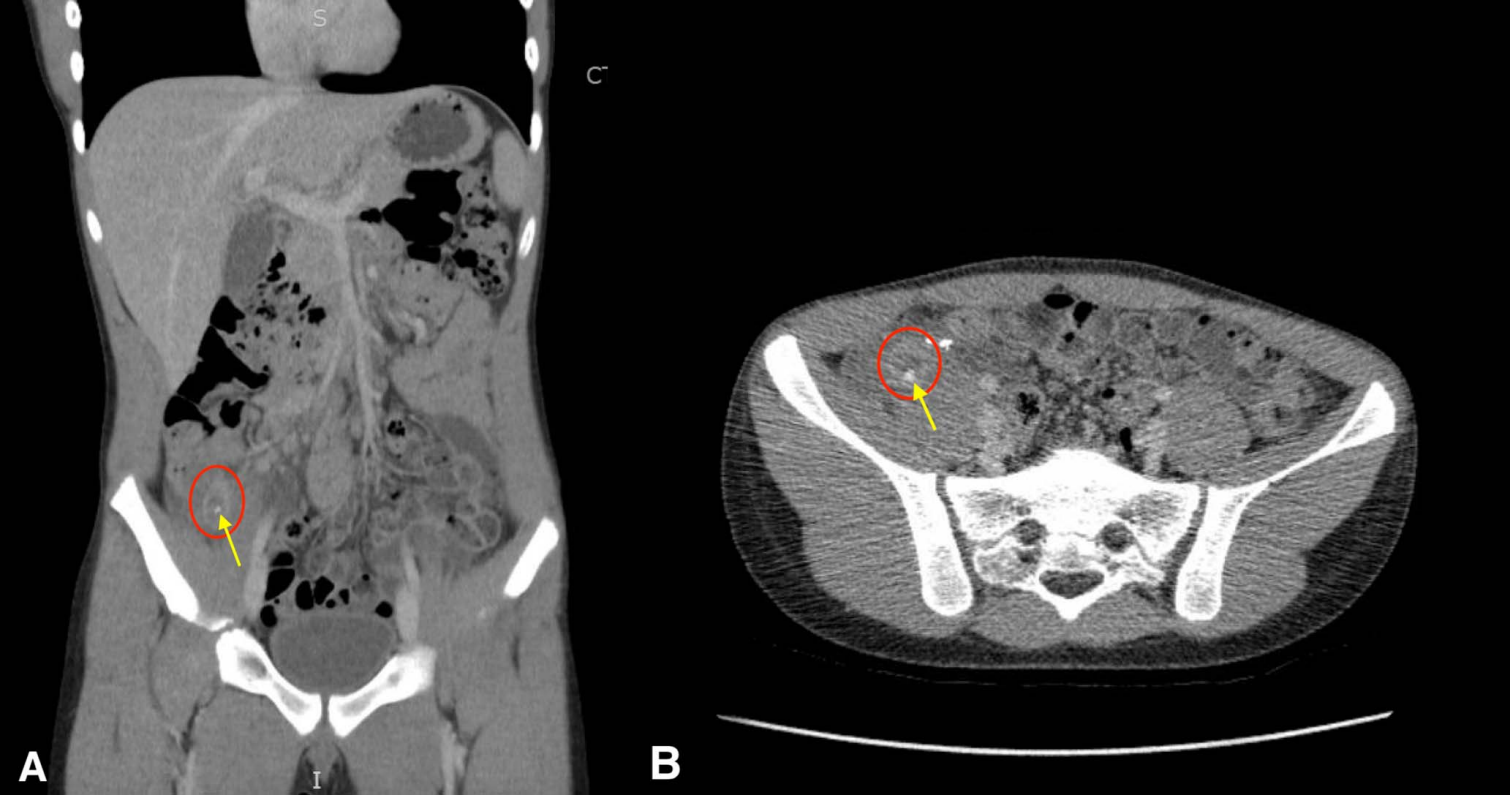
Computed tomography with contrast (A) coronal and (B) axial views of the abdomen and pelvis show the inflamed structure in the region of the previous appendectomy with possible fecalith. The red circles surround the lesion, and the yellow arrows point to fecalith.

Because of concern for a potential abscess and the possible spread of infection if the patient underwent immediate operative management, the decision was made to initially treat the patient nonoperatively with a course of antibiotics and plan an elective diagnostic laparoscopy after 6 to 8 weeks. He was started on intravenous (IV) ceftriaxone 50 mg/kg once daily and IV metronidazole 7.5 mg/kg every 6 hours, IV ondansetron 0.15 mg/kg every 6 hours as needed (PRN), oral acetaminophen 15 mg/kg every 4 hours PRN, and a clear liquid diet.

During the first 2 days of admission, the patient continued to have intermittent abdominal pain and nausea requiring the PRN medications and developed fevers up to 101.3 °F. He also started having loose stools. Because the patient developed fevers while on IV ceftriaxone and metronidazole, he was switched to IV piperacillin-tazobactam 100 mg/kg every 8 hours on hospital day 2. His fevers resolved and pain improved on day 4. On hospital day 5, after being afebrile for more than 24 hours, the patient was discharged home on oral amoxicillin-clavulanate 45 mg/kg/day divided twice daily and oral metronidazole 10 mg/kg 3 times daily to complete a total 10-day course of antibiotics.

At his follow-up appointment 10 days after discharge, the patient had finished the oral antibiotics and was doing well without any abdominal pain, fever, diarrhea, or change in appetite or activity level. At his 2-month follow-up appointment, the patient remained asymptomatic.

He underwent laparoscopic surgery 9 weeks postdischarge for removal of the appendiceal stump. No signs of residual infection, inflammation, or fecalith were seen during the procedure. The patient had an uneventful postoperative recovery.

## DISCUSSION

First reported by Rose in 1945,^5^ stump appendicitis is a rare but increasingly well-known complication of appendectomy. The incidence of stump appendicitis ranges between 0.06% and 0.15%; however, the true incidence is unknown as the condition is commonly underreported.^2,6,7^ Stump appendicitis occurs in all age groups and can occur anytime between 2 weeks to 60 years after initial appendectomy.^2^ Diagnosis of stump appendicitis can be difficult given the paradoxical history of symptoms similar to acute appendicitis despite the patient being status post appendectomy. While abdominal ultrasound can be used to screen for stump appendicitis, abdominal CT with contrast is usually required for definitive diagnosis and has become the gold standard.^8^ Risk factors for stump appendicitis include history of complicated acute appendicitis at initial appendectomy and appendiceal stump length >0.5 cm.^3^ A systematic review by Casas et al showed that 80% of stump appendicitis cases occurred in patients with a history of complicated acute appendicitis such as suppurative, gangrenous, or perforated acute appendicitis.^3^ Casas et al hypothesized that severe inflammation and adherence of the appendix to surrounding tissues can hinder the view of the appendiceal base, thus increasing the risk of leaving a longer appendiceal stump.^3^ Casas et al suggested that residual appendiceal stump length after appendectomy should be no longer than 0.5 cm because they found no cases of stump appendicitis with a stump length <0.5 cm, and any greater length could allow fecalith impaction.^3^ A common assumption is that the risk of stump appendicitis is higher with initial laparoscopic appendectomy compared to open appendectomy because of the narrow 2-dimensional view and lack of tactile feedback.^3,8-10^ However, the literature shows no evidence that laparoscopic appendectomy increases the risk of stump appendicitis compared to open appendectomy, and Casas et al reported that the majority of stump appendicitis cases followed open appendectomy.^3^ The reason for higher rates of stump appendicitis after open appendectomy is unknown; however, Paudyal et al hypothesized that it is because laparoscopy is a relatively new technology and more cases of open appendectomy have been performed and reported historically.^4^ The incidence of stump appendicitis cases following laparoscopic appendectomy has increased since 1990 and is expected to continue to increase as laparoscopic techniques become more prevalent.^3^

Our case describes a clinical presentation of stump appendicitis in a pediatric patient and highlights initial nonoperative management, which is not well reported in the literature. A literature search of PubMed and Google Scholar revealed 27 reported cases of stump appendicitis in patients <18 years, and only 2 cases were managed nonoperatively with antibiotics and symptom management.^11,12^ No guidelines for treatment of stump appendicitis are currently available, and our case highlights the potential for successful initial nonoperative management in this population.

Common concerns for nonoperative management of appendicitis are the efficacy of antibiotics and the risks involved with nonoperative treatment. In addition to the 2 pediatric cases reporting nonoperative management, we found a case series reporting the successful management of stump appendicitis with IV antibiotics and bowel rest in 2 adults.^4,11,12^ The patients had no recurrence of symptoms at follow-up, which ranged between 2 weeks and 9 months.

Although the evidence in stump appendicitis treatment is limited, studies show that the efficacy of nonoperative management for pediatric acute appendicitis is 78% to 92%.^13,14^ One study reported 67% efficacy, but a significant number of patients were lost to follow-up, decreasing the strength of this result.^15^ Whether the results for acute appendicitis can be extrapolated to stump appendicitis is unclear, but studies show evidence of high efficacy of nonoperative treatment of appendicitis in the pediatric population. In addition, many cases that required surgical intervention of stump appendicitis described peritonitis, perforation, and gangrenous tissue.^8-10^ Thus, initial nonoperative management is most suitable for the select population with uncomplicated disease.

The potential risks of nonoperative treatment include possible progression of disease, recurrence, adverse effects of antibiotics, and increased hospital length of stay.^13^ In a systematic review comparing nonoperative and operative management of pediatric acute appendicitis during the coronavirus disease 2019 (COVID-19) lockdown period, 11.5% of nonoperative patients developed complications such as abscesses, perforation, gangrene, and disease recurrence vs 3.1% of operative patients.^13^ Although higher rates of negative outcomes were associated with conservative management, the COVID-19 pandemic may have contributed to the higher rates of negative outcomes by causing delayed presentation, diagnosis, and treatment.^16^

Side effects of antibiotics used to treat stump appendicitis are usually mild and self-limiting once the course is finished,^17^ as seen in our patient who had loose stools that had resolved at follow-up. The other cases of conservative management for stump appendicitis did not report any adverse effects or negative outcomes.^4,11,12^ The evidence for hospital length of stay with conservative management of appendicitis compared to appendectomy is mixed and needs to be further evaluated.^13,18^ Thus, although nonoperative treatment of stump appendicitis has potential risks, they may be less significant than one might expect. However, studies need to be done to elucidate them further.

An interesting difference between our patient and the reports in the literature is the choice of antibiotics used to treat stump appendicitis. In both children and adults, IV third-generation cephalosporins—such as ceftriaxone, cefotaxime, or cefoperazone—plus metronidazole were used in the hospital, and the patients were discharged on an oral second- or third-generation cephalosporin and metronidazole.^4,11,12^ One pediatric patient was placed on additional gentamicin while in the hospital, and his oral antibiotics are unknown.^11^ While our patient was started on similar medications, the team broadened his antibiotic coverage from IV ceftriaxone and metronidazole to IV piperacillin-tazobactam because he did not show immediate clinical improvement as expected. Possibly, our patient simply needed more time on his initial IV regimen to show improvement; *Pseudomonas* species and multidrug-resistant bacteria were unlikely to have been involved in our case. Another difference between our case and reported cases of nonoperative management of stump appendicitis is the oral antibiotic regimen of choice upon discharge from the hospital. Our patient was discharged on oral amoxicillin-clavulanate and metronidazole instead of a third-generation cephalosporin and metronidazole. All of these regimens (IV third-generation cephalosporins + metronidazole, IV piperacillin-tazobactam ± gentamicin, and IV amoxicillin-clavulanate ± gentamicin) are recommended by global surgical and infectious diseases societies as first-line antibiotic therapy in treatment of acute appendicitis and may be options for treatment of stump appendicitis as well.^19^ The efficacy and risks of the different regimens in stump appendicitis, however, need to be further studied before a general guideline can be made.

The benefits of nonoperative management include avoiding the potential risks of surgery, especially repeat procedures, and decreased health care costs. Risks of surgery include bleeding, infection, and injury to surrounding structures.^13^ Cost-benefit simulation analyses of uncomplicated acute appendicitis in the pediatric population in 2017 and 2023 demonstrated a decreased cost of $2,300 to $2,700 USD per patient with nonoperative management compared to surgical appendectomy.^20,21^ However, whether these data can be extrapolated to the treatment of stump appendicitis is unknown, so cost-benefit analyses are required.

Stump appendicitis is commonly believed to be a progressive disease if not treated surgically.^8^ However, our case and the literature suggest that stump appendicitis may be treated successfully with nonoperative management of IV antibiotics and symptom management in select patients.

## CONCLUSION

We report a case of stump appendicitis in a child who was managed nonoperatively in the hospital. Although surgical intervention is the principal form of management, our case suggests that initial conservative management may be considered in select cases. Further studies are needed to evaluate the efficacy, benefits, and risks of treating acute stump appendicitis with antibiotics and symptom management in this patient population before general guidelines can be made. Our case highlights the importance of considering stump appendicitis as a possible cause of recurrent abdominal pain in children with a history of appendectomy and the potential for nonoperative management in select cases.

## Notes

*This article meets the Accreditation Council for Graduate Medical Education and the American Board of Medical Specialties Maintenance of Certification competencies for Patient Care and Medical Knowledge.*
